# NOTCH3 limits the epithelial–mesenchymal transition and predicts a favorable clinical outcome in esophageal cancer

**DOI:** 10.1002/cam4.3933

**Published:** 2021-05-27

**Authors:** Norihiro Matsuura, Koji Tanaka, Makoto Yamasaki, Kotaro Yamashita, Takuro Saito, Tomoki Makino, Kazuyoshi Yamamoto, Tsuyoshi Takahashi, Yukinori Kurokawa, Kiyokazu Nakajima, Hidetoshi Eguchi, Hiroshi Nakagawa, Yuichiro Doki

**Affiliations:** ^1^ Department of Gastroenterological Surgery Graduate School of Medicine Osaka University Suita Osaka Japan; ^2^ Herbert Irving Comprehensive Cancer Center Columbia University New York NY USA

**Keywords:** chemotherapy, epithelial–mesenchymal transition, esophageal cancer, Notch signaling, NOTCH3

## Abstract

**Background:**

Esophageal squamous cell carcinoma (ESCC) is the deadliest of all human squamous cell carcinomas and is characterized by chemotherapy resistance and poor prognosis associated with the epithelial–mesenchymal transition (EMT). A subset of ESCC displays loss‐of‐function mutations in genes encoding Notch receptor family members, including NOTCH3. Although Notch signaling regulates EMT in ESCC cells, the role of NOTCH3 in EMT and chemotherapy resistance remains elusive. This study aimed to examine the role of NOTCH3 in EMT and chemotherapy resistance, and determine whether NOTCH3 expression can be used to predict the response to chemotherapy.

**Methods:**

In vitro and in vivo assays were conducted to clarify the contribution of NOTCH3 to chemotherapy resistance. Using specimens from 120 ESCC patients treated with neoadjuvant chemotherapy, we compared the expression levels of NOTCH3 and genes involved in EMT according to the degree of chemotherapy sensitivity.

**Results:**

In ESCC cells, chemotherapy resistance was associated with NOTCH3 downregulation and concurrent activation of EMT. RNA interference to silence NOTCH3 resulted in induction of the EMT marker Vimentin (VIM), leading to chemotherapy resistance in ESCC cells. Conversely, ectopic expression of the activated form of NOTCH3 suppressed EMT and sensitized cells to chemotherapy. Results of chromatin immunoprecipitation assays suggested that NOTCH3 may repress transcription of the VIM.

**Conclusions:**

Our findings suggest that NOTCH3 may control chemotherapy sensitivity by regulating EMT. NOTCH3 may serve as a novel biomarker to predict better clinical outcomes in ESCC patients.

## Graphical abstract

Notch3 may control chemotherapy sensitivity by regulating the epithelial‐mesenchymal transition. Notch3 may predict better clinical outcomes in esophageal squamous cell carcinoma patients.

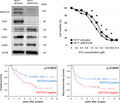

## 1 INTRODUCTION

Esophageal squamous cell carcinoma (ESCC) is one of the most aggressive and lethal malignancies.^1^ Esophagectomy is the standard treatment for resectable ESCC, but systemic and local recurrence can occur even after curative resection.^2^, ^3^, ^4^ To improve prognosis, a combination of preoperative chemotherapy or chemoradiotherapy (i.e., neoadjuvant chemotherapy; NAC) followed by surgery is performed. The effects of preoperative chemotherapy differ between patients, and the prognosis of patients for whom these treatments are ineffective is poor. Clarification of the mechanism of resistance to chemotherapy is an important goal in considering strategies to improve the prognosis of ESCC patients.

Cancer cells can activate diverse signaling pathways and acquire chemotherapy resistance to evade drug cytotoxicity.^5^, ^6^ Studies have shown that chemotherapy resistance can be induced by the epithelial–mesenchymal transition (EMT) in ESCC.^7^, ^8^, ^9^ In general, EMT is a cellular process in which cells lose their epithelial characteristics and acquire mesenchymal features.^10^ In cancer, EMT is associated with tumorigenesis, invasion, metastasis, tumor stemness, and resistance to stressors such as anticancer drugs, radiation, and hypoxia.^11^, ^12^, ^13^

The Notch pathway regulates cell fate and differentiation processes in a context‐dependent manner. Once Notch receptor, which consists of four types of transmembrane receptors (NOTCH1 to 4), is bound by its ligand, the translocation of the intracellular domain of NOTCH (ICN; the activated form of NOTCH) into the nucleus is triggered. ICN forms a transcriptional activator complex with transcriptional factors such as Recombination Signal Binding Protein for Immunoglobulin Kappa J (RBPJ), and regulates the expression of target genes such as the HES/HEY family.^14^ We previously reported that Notch signaling is important for esophageal epithelial differentiation because ICN1 transcriptionally activates *NOTCH3* to drive squamous differentiation.^15^ We also reported that the expression of NOTCH3 induced by ZEB1 is reduced during EMT, but had not determined whether NOTCH3 inactivation is dispensable for the induction of EMT in ESCC.^16^ A large‐scale genomic analysis of Japanese ESCC patients found that *NOTCH1* and *NOTCH3* harbored mutations at a high frequency (19% and 8%, respectively), and that these mutations often resulted in a loss of function.^17^ While increased NOTCH1 expression is associated with a poor prognosis and/or resistance to treatment in cholangiocarcinoma cancer, ovarian cancer, and ESCC, the role of NOTCH3 in cancer is less well understood.^9^, ^18^, ^19^

The purpose of this study was to determine the contribution of NOTCH3 to chemotherapy resistance and clinical outcomes in ESCC patients. Our findings suggest that NOTCH3 downregulation may reverse the transcriptional repression of *VIM* to facilitate EMT in ESCC cells, leading to chemotherapy resistance and poor prognosis in ESCC patients.

## 2 MATERIALS AND METHODS

### 2.1 ESCC cell lines and chemical reagents

Human ESCC cell lines TE6 (RCB1950) and TE11 (RCB2100) were grown in RPMI‐1640 medium (Nakalai Tesque) supplemented with 10% FBS (Sigma‐Aldrich) in a humidified atmosphere of 5% CO_2_ at 37°C. TE11 derivatives expressing doxycycline‐inducible ICN3, the activated form of NOTCH3 (TE11‐ICN3), or an empty control vector (TE11*) have been described previously.^20^ ICN3 was induced by incubating cells with 1 µg/ml doxycycline (DOX, 631311, Clontech Laboratories) for 24 h. A 5FU‐resistant TE11 derivative (TE11‐FR) was established by passaging TE11 cells at least 10 times in the continuous presence of 3 µM fluorouracil (5‐FU, 068‐01403) for more than 2 months. Cell survival and the half‐maximal inhibitory concentration (IC_50_) for 5FU were determined by WST‐8 assay as described in supplementary materials and method.

### 2.2 ESCC xenograft tumors

Animal studies were performed following a protocol approved by the Ethics of Animal Experiments Committee of Osaka University. For xenograft models, TE11* and TE11‐ICN3 (3.0 × 10^6^) cells were suspended in 100 μl RPMI 1640/Matrigel (Becton, Dickinson and Company) and subcutaneously injected into 8‐week‐old female mice (BALB/c‐nu/nu; CLEA). Tumor volume was measured with calipers and calculated using the formula *V* = (*ab* ^2^)/2, where *a* is the smallest diameter and *b* is the largest diameter. To induce ectopic ICN3 expression in vivo, DOX was administered to mice via drinking water (1 mg/ml in 5% sucrose) starting from Day 24 after xenograft transplantation. When the average tumor size reached 100 mm^3^, 5‐FU (5 mg/kg) or PBS (vehicle control) was administered every 3 days by intraperitoneal injection for 21 days. Mice were euthanized on day 21 and tumors were collected. The tumors were fixed in 10% buffered formalin for immunohistochemistry, or lysed for Western blot analysis.

### 2.3 ESCC patients

A total of 120 ESCC patients underwent esophagectomy following NAC at Osaka University Medical Hospital from January 2010 to December 2014 in a study performed under the Institutional Review Board‐approved protocol (08226‐13) in accordance with the Declaration of Helsinki. Patients received one of two regimens as NAC. The first regimen included adriamycin, cisplatin, and fluorouracil (ACF; adriamycin 35 mg/m^2^ and cisplatin 70 mg/m^2^ i.v. on day 1, and fluorouracil 700 mg/m^2^ continuous infusion for 5 days) every 4 weeks. The second regimen included docetaxel, cisplatin, and fluorouracil (DCF; docetaxel 70 mg/m^2^ and cisplatin 70 mg/m^2^ i.v. on day 1, and fluorouracil 700 mg/m^2^ continuous infusion for 5 days) every 3 weeks.^1^ Data on patient characteristics, histologic examination, and survival were obtained from medical charts. Therapeutic effect was evaluated according to the histological criteria set forth by the Japanese Society of Esophageal Disease.^21^ Briefly, therapeutic efficacy was divided into five categories (grade 0, 1a, 1b, 2, or 3) based on the proportion of the tumor affected by degeneration or necrosis. Patients underwent regular follow‐up for 4–8 weeks after surgery, and were then assessed every 3 months during the first 2 years, every 6 months for the subsequent 3 years, and then annually from 5 years after surgery. Radiological investigations, usually by CT, were performed when there was a suspicion of recurrent disease or an endoscopic finding. All recurrences were confirmed by histological or radiological examination. Patient status was recorded at the last visit for survival analysis.

### 2.4 Chromatin immunoprecipitation (ChIP) assay

To perform ChIP assays, 2 × 10^6^ cells grown for 22 h in 100‐mm dishes were treated with 1% formaldehyde for 10 min at 37°C and quenched with 0.125 M glycine for 5 min at room temperature. Cross‐linked chromatin was sheared into ~500 bp DNA fragments with Covaris S220 (M&S Instruments Inc.). Sheared chromatin (20 μg) was incubated for immunoprecipitation with an antibody against NOTCH3 (#2889, dilution 1:50, Cell Signaling Technology) or RBPJ (ab25949, dilution 1:100, Abcam), or negative control mouse IgG (53010, dilution 1:10, ACTIVE MOTIF). DNA was purified with the Chromatin IP DNA purification kit (58002, ACTIVE MOTIF) and analyzed by real‐time qPCR using THUNDERBIRD^®^ SYBR^®^ qPCR Mix and the Applied Biosystems 7900HT Fast Real Time PCR system (Thermo Fisher Scientific). The following primers were used for real‐time qPCR: 5’‐AGCTGCAGGCGCTAGTTG‐3’ and 5’‐CACACCCAAACACCACGTATT‐3’ for RBPJ‐binding sites in the 2nd intron of *VIM*, and 5’‐TTTGCCGTGATATATAGGATAATTT‐3’ and 5’‐TGATGCTGAGAAGTTTCGTTG‐3’ for an off‐target control region of *VIM*, lacking RBPJ binding sites. Data represent at least three independent experiments.

### 2.5 Immunohistochemistry and TdT‐mediated dUTP nick end labeling (TUNEL) assay

Tumor specimens were fixed with 10% formalin, and paraffin‐embedded tissue blocks were sectioned into 3.5‐μm slices. The sections were deparaffinized in xylene and dehydrated in a graded ethanol series. For antigen retrieval, sections were incubated in 10 mM citrate buffer at 110°C using a pressure cooker for 15 min. Endogenous peroxidase activity in the tissue specimens was blocked by incubating the slides in 3% hydrogen peroxide (H_2_O_2_) solution in methanol at room temperature for 20 min. After treatment of the sections with 1% horse serum albumin for 30 min at room temperature to block nonspecific reactions, all sections were incubated with primary antibodies in a humidified chamber at 4°C overnight. Antibodies used included anti‐NOTCH3 polyclonal antibody (ab23426, dilution 1:300, Abcam), anti‐E‐cadherin (CDH1) monoclonal antibody (#3195, dilution 1:100, Cell Signaling Technology), anti‐N‐cadherin (CDH2) polyclonal antibody (ab18203, dilution 1:300, Abcam), and anti‐Vimentin (VIM) monoclonal antibody (#5741, dilution 1:300, Cell Signaling Technology). After incubation with secondary antibodies for 20 min at room temperature, the reactions were visualized using VECTASTAIN^®^ Elite^®^ ABC Kit (PK‐6100, VECTOR LABORATORIES), which stains the targeted antigen brown, and hematoxylin counterstaining. Two investigators (N. M and K. T) independently evaluated the stained sections. The grade and area of nuclear staining with the anti‐NOTCH3 antibody of cells remaining after NAC were evaluated and divided into two groups: NOTCH3‐positive and NOTCH3‐negative. The degree of CDH2 and VIM staining was evaluated based on the extent of membranous staining.

TUNEL assays were performed to evaluate apoptosis in formalin‐fixed, paraffin‐embedded xenograft tumor tissue samples. In brief, paraffin sections (3.5 μm) were deparaffinized with xylene and then rehydrated in a graded alcohol series. TUNEL signal was detected using the ApopTag Fluorescein In Situ Apoptosis Detection Kit (Chemicon International). Nuclei were counterstained using VECSTASHIELD Mounting Medium with DAPI (VECTOR Laboratories). Green fluorescence from apoptotic cells was analyzed with a fluorescence microscope (BZ‐X 710; KEYENCE). TUNEL‐positive cells were considered apoptotic cells.

### 2.6 Statistical analysis

Each experiment was repeated three times. Data are expressed as mean ± SD. Mean values were compared using Student's *t*‐test. In vivo tumor growth was analyzed with one‐way ANOVA for repeated measures. Discrete variables were assessed with the *χ* ^2^‐test. Overall survival (OS) was defined as the time interval between the day of surgery and day of death or last follow‐up. Recurrence‐free survival (RFS) was defined as the time interval between the day of surgery and documented date of the first recurrence. Survival was calculated according to the Kaplan–Meier method and compared by the log‐rank test. *p* < 0.05 was considered statistically significant. Statistical analyses were performed using JMP Pro 14.0 (SAS Institute).

## 3 RESULTS

### 3.1 5‐FU decreases NOTCH3 expression while inducing EMT in surviving ESCC cells

**FIGURE 1:**
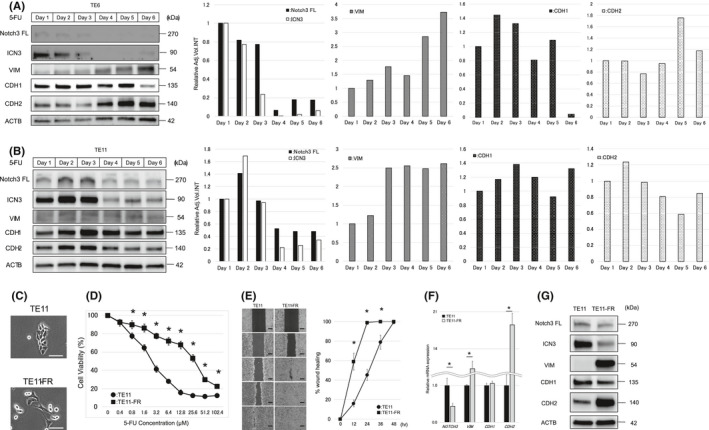
NOTCH3 and EMT marker modulation by short‐ and long‐term exposure to 5‐FU. (A and B) Expression of NOTCH3 FL, ICN3, VIM, CDH1, and CDH2 upon exposure to 20 µM 5‐FU, as assessed by Western blot, in (A) TE6 and (B) TE11 cells. In densitometry, the signal intensity for the molecule of interest was calibrated by that of ACTB at each time point. The relative expression was expressed compared to the signal intensity at day 1 as 1. Cell lysates were prepared every 24 h after 5‐FU exposure. (C) Morphology of TE11‐FR cells. Scale bars, 100 μm. (D) 5‐FU sensitivity assay in TE11‐FR cells. (E) Wound healing assay in TE11 and TE11‐FR cells. Scale bars, 500 μm. (F) Expression of *NOTCH3*, *VIM*, *CDH1*, and *CDH2* mRNA, as assessed by reverse transcription (RT)‐qPCR, in TE11 and TE11‐FR cells. (G) Expression of NOTCH3 FL, ICN3, VIM, CDH1, and CDH2, as assessed by Western blot, in TE11 and TE11‐FR cells. NOTCH3 FL: NOTCH3 full length, ICN3: intracellular NOTCH3, TE11‐FR: TE11 5‐FU resistant cell line. ACTB served as a loading control in (A), (B), and (G). *GAPDH* served as an internal control gene in (F)

The expression levels of NOTCH3 and EMT markers VIM (Vimentin), CDH2 (N‐cadherin), and CDH1 (E‐cadherin) in ESCC cell lines were shown in Figure S1. We first examined how ESCC cells react to 20 µM 5‐FU using two ESCC cell lines (TE6 and TE11). Surviving cells of both cell lines gradually became spindle‐shaped (Figure S2A and B), and expression of VIM, a mesenchymal marker, gradually increased, albeit to a modest extent (Figure 1A and B). In TE6 cells, the expression of CDH2 gradually increased and that of CDH1 gradually decreased (Figure 1A). In TE11 cells, the expression of CDH1 and CDH2 did not change markedly (Figure 1B). The expression of full‐length NOTCH3 (NOTCH3 FL) and the activated form of NOTCH3 (ICN3) gradually decreased from about 3 or 4 days after exposure to 5‐FU in both cell lines, in contrast to EMT markers (Figure 1A and B).

### 3.2 Long‐term 5‐FU exposure reduces NOTCH3 expression in mesenchymal ESCC cells

To gain a mechanistic insight into the association between NOTCH3 or EMT markers and chemoresistance, we have first established a TE11 derivative, designated TE11‐FR with an increased 5‐FU resistance. To this end, parental TE11 cells were cultivated in the continuous presence of 3 µM 5‐FU for 8 weeks. While this concentration of 5‐FU killed initially >50% of parental cells within 72 h (Figure 1D), the surviving cells continued proliferation, albeit slower and were successfully passaged >10 times. Resulting TE11‐FR cells displayed spindle‐shaped morphology compared to parental TE11 cells (Figure 1C). A 5‐FU sensitivity assay showed that TE11‐FR cells were more resistant to 5‐FU than TE11 cells (Figure 1D), with IC_50_ values estimated at 2.44 and 26.1 µM, respectively. TE11‐FR cells were also more resistant to other anticancer drugs such as cisplatin and docetaxel than TE11 cells (Figures S2D and E). TE11‐FR cells also showed a greater degree of cell migration (Figure 1E) and had significantly lower *NOTCH3* mRNA expression, higher *VIM*, and *CDH2* mRNA expression compared to TE11 cells (Figure 1F). Similar results were observed by Western blot, with lower NOTCH3 FL and ICN3 expression, higher VIM and CDH2 expression, and lower CDH1 expression in TE11‐FR cells compared to TE11 cells (Figure 1G).

### 3.3 RNAi against NOTCH3 induces EMT and 5‐FU resistance

**FIGURE 2:**
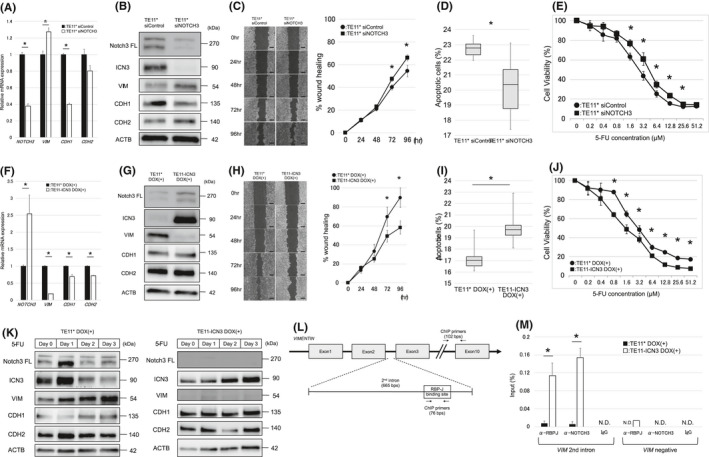
Effects of suppression and activation of NOTCH3 and ChIP assays. (A and B) Expression of NOTCH3 FL, ICN3, VIM, CDH1, and CDH2 following NOTCH3 suppression, as assessed by (A) reverse transcription (RT)‐qPCR and (B) Western blot. (C) Wound healing assay over the course of 4 days after treatment with siRNA against NOTCH3 (siNOTCH3). Scale bars, 500 μm. (D) The proportion of apoptotic cells after exposure to 10 μM 5‐FU for 72 h following treatment with siNOTCH3. (E) 5‐FU sensitivity assay following treatment with siNOTCH3. (F and G) Expression of NOTCH3 FL, ICN3, VIM, CDH1, and CDH2 following ectopic expression of ICN3, as assessed by (F) RT‐qPCR and (G) Western blot. (H) Wound healing assay over the course of 4 days following ectopic ICN3 expression. Scale bars, 500 μm. (I) The proportion of apoptotic cells after exposure to 10 μM 5‐FU for 72 h following ectopic ICN3 expression. (J) 5‐FU sensitivity assay following ectopic ICN3 expression. (K) Expression of NOTCH3 FL, ICN3, VIM, CDH1, and CDH2 after exposure to 20 μM 5‐FU exposure, as assessed by Western blot, in the presence or absence of ectopic ICN3 expression. (L) Schematic of the 2nd intron of *VIM* and primer sequences. (M) ChIP assays to assess the binding of RBPJ and NOTCH3 to the 2nd intron and a negative control region of *VIM* in TE11* and TE11‐ICN3 cells. NOTCH3 FL: NOTCH3 full length, ICN3: intracellular NOTCH3, TE11*: TE11 Tet‐on control cell line, TE11‐ICN3: TE11 Tet‐on ICN3 cell line, DOX: doxycycline, ChIP: chromatin immunoprecipitation. ACTB served as a loading control in (B), (G), and (K). *GAPDH* served as an internal control gene in (A) and (F)

To examine whether NOTCH3 plays an important role in EMT and chemoresistance, NOTCH3 was silenced by RNAi (siNOTCH3). As shown in Figure 2A, siNOTCH3 increased the expression of *VIM* mRNA and decreased the expression of *CDH1* mRNA, as assessed by RT‐qPCR (Figure 2A). At the protein level, siNOTCH3 similarly reduced the expression of ICN3 and CDH1, and increased the expression of VIM and CDH2 (Figure 2B). siNOTCH3 also significantly increased cell migration (Figure 2C).

Apoptosis assays were performed to determine the proportion of apoptotic cells 72 h after exposure to 10 µM 5‐FU. The proportion of apoptotic cells was significantly reduced in siNOTCH3‐treated cells relative to siControl‐treated cells (20.4 ± 1.86% vs. 22.7±0.6%, *p* = 0.02; Figure 2D and Figure S3C). In chemosensitivity assays, siNOTCH3‐treated cells were significantly resistant to 5‐FU treatment compared to siControl‐treated cells, with IC_50_ values of 4.12 and 2.89 μM, respectively (Figure 2E). These results suggest that the down‐regulation of NOTCH3 expression is associated with 5‐FU resistance via the induction of EMT.

### 3.4 Ectopic ICN3 expression increases sensitivity to 5‐FU

To test the effect of ICN3 expression on EMT induced by chemotherapy and response of chemotherapy, TE11* cells and TE11 cells ectopically expressing ICN3 (TE11‐ICN3 cells) were exposed to 1 μg/ml DOX. *VIM* mRNA expression in TE11‐ICN3 cells was lower than that in TE11* cells (Figure 2F). The lower expression of VIM in TE11‐ICN3 cells was confirmed by Western blot, although the expression of CDH2 and CDH1 did not significantly differ between TE11* and TE11‐ICN3 cells (Figure 2G). These results suggest that NOTCH3 activation has a negative impact on VIM expression.

As shown in Figure 2H, migration was also significantly reduced in TE11‐ICN3 cells compared to TE11* cells. Moreover, the proportion of apoptotic cells was significantly increased in TE11‐ICN3 cells compared to TE11* cells (17.3 ± 1.27% vs. 20.0 ± 1.62%, *p* = 0.02; Figure 2I and Figure S3F). In 5‐FU‐sensitivity assays, TE11‐ICN3 cells were more sensitive to 5‐FU than TE11* cells, with IC_50_ values of 1.56 and 2.90 μM, respectively (Figure 2J).

### 3.5 Ectopic ICN3 expression limits EMT induced by 5‐FU

We evaluated temporal changes in the expression of EMT markers following exposure to 20 μM 5‐FU. As 5‐FU exposure time progressed, TE11* cells gradually took on a spindle shape, and most TE11‐ICN3 cells had died by Day 4 (Figure S3G). Western blot showed a gradual decrease in ICN3 expression and increase in VIM expression in TE11* cells, whereas VIM expression did not increase in TE11‐ICN3 cells (Figure 2K). These results suggest that continuous NOTCH3 activation can prevent the increase in VIM expression induced by 5‐FU exposure.

### 3.6 Transcriptional repression of VIM by ICN3

Given the clear negative correlation between the expression of ICN3 and VIM, we next looked into whether the correlation involved a transcriptional aspect. To this end, we analyzed the *VIM* locus and searched for RBPJ binding sites using ChIP‐Atlas.^22^ When Notch signals are transmitted, Notch intracellular domains such as ICN3 do not bind to DNA directly, but rather bind to co‐factor RBPJ, which binds to DNA directly, and transduces the signal. In ChIP‐Atlas, there were no peaks within 20 kb upstream and downstream of the *VIM* locus, except for the 2nd intron region of *VIM* (Figure 2L). ChIP‐PCR revealed that, among DNA fragments immunoprecipitated by an anti‐RBPJ antibody, a substantially greater amount of DNA fragments corresponding to the 2nd intron was immunoprecipitated in TE11‐ICN3 cells compared to TE11* cells. Similar results were obtained using an anti‐NOTCH3 antibody. A negative control primer pair targeting the 9th intron of *VIM* showed no difference in the amount of DNA fragments immunoprecipitated from TE11* and TE11‐ICN3 cells (Figure 2M).

### 3.7 Antitumor effect of 5‐FU in ESCC xenograft mice

**FIGURE 3:**
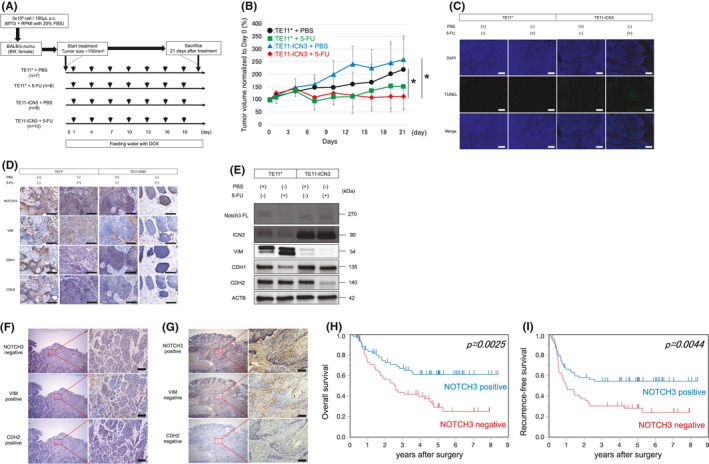
Antitumor effect of 5‐FU in an ESCC xenograft mouse model and immunohistochemical staining of ESCC samples from patients treated with NAC. (A) Summary of the xenograft model using TE11* and TE11‐ICN3 cell lines. Female nu/nu mice (8 weeks of age) were subcutaneously injected with 3 × 10^6^ cells. When the average tumor volume reached approximately 100 mm^3^, mice were provided with water containing DOX. PBS or 5 mg/kg 5‐FU was administered to these mice by intraperitoneal injection every 3 days. (B) Tumor volume in TE11‐ICN3 mice treated with 5‐FU was significantly lower than in corresponding mice treated with PBS. (C) Analysis of apoptosis by TUNEL staining (blue: DAPI staining for nuclei, cyan: TUNEL staining) in tumors of xenograft mice. (D) Immunohistochemical analysis of NOTCH3, VIM, CDH2, and CDH1 in tumors of xenograft mice. Scale bar, 100 μm. (E) Western blot analysis of lysates prepared from tumor tissue of mice treated with vehicle control or 5‐FU for 21 days. ACTB served as a loading control. (F) NOTCH3‐negative, CDH2‐positive, and VIM‐positive IHC staining. Scale bars, 100 μm. (G) NOTCH3‐positive, CDH2‐negative, and VIM‐negative IHC staining. Scale bars, 100 μm. (H and I) Survival curves based on NOTCH3 expression in ESCC patients treated with NAC. (H) Overall survival and (I) recurrence‐free survival. Blue and red lines correspond to NOTCH3‐positive and NOTCH3‐negative groups, respectively. TE11*: TE11 Tet‐on control cell line, TE11‐ICN3: TE11 Tet‐on ICN3 cell line, DOX: Doxycycline, ICN3: intracellular NOTCH3, NAC: neoadjuvant chemotherapy

We next evaluated whether NOTCH3 activation impacts the therapeutic effect of 5‐FU, or whether it inhibits the induction of EMT in ESCC in vivo. A schematic of the experiment is shown in Figure 3A. With respect to tumor growth, tumors were smaller in TE11* xenograft mice (TE11* group) treated with 5‐FU relative to those treated with PBS, although the difference was not significant (152.0 ± 94.4% vs. 219.3 ± 134.2%, *p* = 0.22). However, tumors in TE11‐ICN3 xenograft mice (TE11‐ICN3 group) treated with 5‐FU were significantly smaller than in corresponding mice treated with PBS (111.3 ± 49.4% vs. 258.8 ± 83.5%, *p* = 0.0016). Compared to mice treated with PBS, those treated with 5‐FU showed less tumor growth in both TE11* and TE11‐ICN3 groups, although the difference was only significant in the TE11‐ICN3 group (Figure 3B). TUNEL assay revealed that 5‐FU induced a higher degree of apoptosis in the TE11‐ICN3 group compared to the TE11* group (Figure 3C).

We next evaluated cells that remained after 5‐FU treatment by immunohistochemistry (Figure 3D). In the TE11* group, nuclear staining for NOTCH3 in tumor cells was modest in mice treated with PBS, and absent in those treated with 5‐FU. In contrast, in the TE11‐ICN3 group, nuclear staining was observed in tumor cells of both mice treated with PBS and 5‐FU. In tumor cells of the TE11* group, VIM expression was observed only in mice treated with 5‐FU, whereas it was absent in mice treated with either PBS or 5‐FU in the TE11‐ICN3 group. Similar results were observed with CDH2 staining. CDH1 was present in tumor cells of both groups under PBS and 5‐FU treatment conditions.

Western blot showed that NOTCH3 FL and ICN3 were less detectable in mice treated with 5‐FU compared to those treated with PBS in the TE11* group (Figure 3E), whereas no difference was observed in ICN3 levels in mice treated with PBS or 5‐FU in the TE11‐ICN3 group. VIM expression in mice treated with 5‐FU in the TE11* group was higher than that in mice treated with PBS, whereas in the TE11‐ICN3 group, it was lower in mice treated with 5‐FU than in those treated with PBS. CDH1 expression in mice treated with 5‐FU was lower than in those treated with PBS in the TE11* group, and CDH2 expression was lower in mice treated with 5‐FU than in those treated with PBS in the TE11‐ICN3 group.

### 3.8 NOTCH3, CDH2, and VIM expression in ESCC patients after NAC by immunohistochemistry

Patient characteristics are summarized in Table 1. Of the 120 samples analyzed in the present study, 57 showed weak expression of NOTCH3 in residual tumors. In samples with weak NOTCH3 expression (NOTCH3‐negative group), pathologic stage was more advanced. We next evaluated the correlation between NOTCH3 expression and CDH2 or VIM expression in ESCC tissue. The expression of CDH2 and VIM was higher in the NOTCH3‐negative group compared to the NOTCH3‐positive group (Figure 3F and G). Table 2 summarizes correlations between the therapeutic effect of NAC and the expression of NOTCH3, CDH2, and VIM. Samples that were NOTCH3‐negative, CDH2‐positive, or VIM‐positive were correlated with poor therapeutic efficacy of NAC. The median follow‐up period among survivors was 1822.5 days (range, 138–3061). Five‐year OS and RFS rates were 29.1% and 28.2%, respectively, in the NOTCH3‐negative group, and 61.1% and 54.6%, respectively, in the NOTCH3‐positive group, with a significant difference between the two groups (log‐rank test: *p* = 0.0025 and 0.0044, respectively) (Figure 3H and I).

**TABLE 1:** Correlation between NOTCH3 expression and various clinicopathological parameters in ESCC patients receiving NAC

		NOTCH3‐positive (*n* = 63)	NOTCH3‐negative (*n* = 57)	*p*‐value
Age	Median (range)	68 (38–82)	66 (49–82)	0.37
Sex	Male/female	57/6	54/3	0.38
Location	Ut/Mt/Lt	14/26/23	8/29/20	0.42
pTstage	1/2/3/4	9/16/38/0	7/8/40/2	0.20
pNstage	0/1/2/3	24/23/9/7	13/26/14/4	0.17
pMstage	0/1	56/7	47/10	0.31
pStage^a^	I/II/III/IV	11/20/25/7	2/14/31/10	0.04
Differentiation	por/mod/wel^b^	4/25/8	7/26/7	0.67
Lymphatic invasion	(−)/(+)	15/48	8/49	0.17
Venous invasion	(−)/(+)	40/23	33/24	0.53
VIM expression	(−)/(+)	40/23	13/44	<0.001
CDH2 expression	(−)/(+)	52/11	35/22	0.0096

^a^ pStage was evaluated by UICC 7th edition.

^b^ por/mod/wel: poorly/moderately/well.

**TABLE 2:** Correlation between NOTCH3, VIM, or CDH2 expression and therapeutic effect grade in ESCC patients receiving NAC

		Therapeutic effect		*p*‐value
		Grade 0, 1a, 1b	Grade 2	
NOTCH3	Positive (*n* = 63)	49 (77.8%)	14 (22.2%)	0.04
	Negative (*n* = 57)	52 (91.2%)	5 (8.8%)	
VIM	Positive (*n* = 67)	62 (92.5%)	5 (7.5%)	0.007
	Negative (*n* = 53)	39 (73.6%)	14 (26.4%)	
CDH2	Positive (*n* = 33)	32 (97.0%)	1 (3.0%)	0.005
	Negative (*n* = 87)	69 (79.3%)	18 (20.7%)	

## 4 DISCUSSION

In this study, we discovered that NOTCH3 functions as a key repressor of EMT. While EMT was induced following 5‐FU exposure and subsequent NOTCH3 suppression in ESCC cells, continuous expression of ectopic ICN3 suppressed EMT and increased the sensitivity of tumor cells to 5‐FU. Mechanistically, ICN3 binds to the 2nd intron of *VIM* in a complex with RBPJ, suggesting that it may directly regulate VIM expression. These findings provide new mechanistic insight into how VIM expression is regulated in ESCC in response to chemotherapy.

We focused on NOTCH3 given its opposite regulation during differentiation and EMT. For instance, normal esophageal epithelium differentiates via the sequential activation of NOTCH1 and NOTCH3.^20^, ^23^ In contrast, when TGF‐β induces EMT in ESCC, NOTCH3 is suppressed.^16^ Thus, NOTCH3 appears to function like a switch in decisions to differentiate or induce EMT. In the present study, NOTCH3 FL and ICN3 expression was gradually decreased, and VIM expression was gradually increased upon exposure to 5‐FU. Silencing of *NOTCH3* expression by RNA interreference induced EMT and rendered ESCC cells resistant to 5‐FU. Thus, chemotherapy induced EMT by suppressing NOTCH3 during the process of acquiring chemoresistance. In a previous study, NOTCH1 was reported to be involved in acquiring resistance to 5‐FU in ESCC.^9^ However, no study to date has reported on the involvement of NOTCH3 in the development of chemoresistance. We considered two potential mechanisms by which NOTCH3 might be implicated. First, 5‐FU altered cells to take on a mesenchymal phenotype by modulating NOTCH3 expression in order to survive, and this modulation led to chemoresistance. Second, mesenchymal cells survived and epithelial cells died upon 5‐FU treatment because cancer cells were heterogenous and comprised both epithelial cells and mesenchymal cells.^24^ Regardless of which mechanism might be involved, 5‐FU suppressed NOTCH3 expression, and this led to EMT and caused cells to acquire a 5‐FU‐resistant mesenchymal phenotype. In response to 5‐FU, EMT was not as robustly induced in parental TE11 cells as TE11‐FR cells that displayed more apparent and sustained EMT characteristics (Figure 1). This may be accounted for by more heterogenous nature of parental TE11 cells than 5‐FU selected TE11‐FR cells. It was also plausible that parental TE11 cells may resist 5‐FU toxicity via more diverse mechanisms beyond EMT. In fact, a TE11 subclone resisting 5‐FU via autophagy and CD44 expression has been described.^25^ Additionally, a previous report suggested that 5‐FU resistance could be explained by factors such as *DPYD* gene amplification.^26^ Thus, it may be informative to further investigate the *DPYD* gene in TE11‐FR cells and their potential resistance to other anti‐cancer drugs, such as cisplatin and docetaxel.

We also investigated whether continuous ICN3 expression would inhibit the acquisition of 5‐FU resistance resulting from the suppression of NOTCH3 expression during exposure to 5‐FU. Both in vitro and in vivo, we found that ectopic ICN3 expression not only reversed the effects of NOTCH3 suppression but also re‐sensitized cells to 5‐FU, and cells morphologically changed to take on a more epithelial phenotype. This finding is consistent with a previous study that reported that NOTCH3 activation inhibited EMT in breast cancer.^27^ Similar to our findings, that study reported that NOTCH3 suppression induced VIM expression and EMT. We also found that ICN3 expression was always negatively correlated with VIM expression. According to previous studies, VIM can promote cell motility and migration,^28^, ^29^, ^30^ reduce CDH1 expression, and activate Snail1. VIM is also useful as a mesenchymal marker.^10^ Based on these findings, the direct regulation of VIM expression by ICN3 suggests that NOTCH3 may play an important role in EMT.

Selective activation of NOTCH3 signaling by ICN3 expression inhibited the acquisition of 5‐FU resistance. Non‐specific inhibitors of Notch, such as gamma‐secretase inhibitors (GSIs), have been used in previous studies, as well as clinical trials.^31^ However, while GSIs exhibit anti‐cancer effects, they are not specific for a particular Notch receptor, inhibit other signaling pathways, and cause intestinal toxicity, likely due to dual inhibition of NOTCH1 and NOTCH2.^32^ Thus, in order to avoid the acquisition of chemoresistance without intestinal toxicity, selective activation of NOTCH3 may be an option. In this regard, it was recently shown that a mutation in *NOTCH3* causes cerebral autosomal‐dominant arteriopathy with subcortical infarcts and leukoencephalopathy (CADASIL), and NOTCH3 selective agonists were suggested to be potentially effective in treating the disease.^33^ Similarly, selective NOTCH3 activation may also be an option for treating ESCC.

We also investigated the transcriptional regulation of VIM by NOTCH3 since VIM expression was correlated with changes in NOTCH3 expression. As mentioned previously, ICN3 translocates into the nucleus and forms a transcriptional activator complex with RBPJ. We first determined whether ICN3 binding sites were present in the *VIM* locus using ChIP‐atlas,^22^ but none were present. We further assessed whether RBPJ binding sites were present, and found no such sites in the promoter region, although a potential binding site was present in the 2nd intron of the *VIM* gene. Using ChIP‐PCR, we found that both RBPJ and ICN3 were bound to the 2nd intron of *VIM*, suggesting that RBPJ is recruited to this locus upon translocation of ICN3 into the nucleus. While previous studies have shown that RBPJ is recruited to transcriptional regulatory sites of target genes by ICN3,^34^ and that RBPJ functions as a transcriptional activator when associated with ICN3,^35^, ^36^ our findings suggest that the complex of ICN3 and RBPJ suppresses VIM expression. In this respect, our findings are more consistent with studies reporting on transcription factors that suppress target gene expression in the Notch pathway.^14^, ^15^ Further studies will be needed to gain a better understanding of the transcriptional regulation of VIM by NOTCH3, and whether other Notch family members have a similar effect on VIM expression, since they all bind to DNA when complexed with RBPJ.

In conclusion, 5‐FU suppresses NOTCH3 expression, leading to EMT and 5‐FU resistance. This suggests that selective activation of NOTCH3 may prevent the acquisition of 5‐FU resistance, possibly via the regulation of VIM expression by ICN3.

## CONFLICT OF INTEREST

The authors have no conflict of interest.

## ETHICAL APPROVAL

This study was approved by the appropriate institutional review boards of Osaka University Hospital (approval number: 08226‐13) and was conducted in accordance with the Declaration of Helsinki.

## Supporting information

Fig S1

Fig S2

Fig S3

Supplementary Material

## Data Availability

The data that support the findings of this study are available from the corresponding author, KT, upon reasonable request.
