# Supplementary material for: NOTCH3 limits the epithelial–mesenchymal transition and predicts a favorable clinical outcome in esophageal cancer
Source: Cancer Med. 2021 May 27;10(12):3986–96. doi: 10.1002/cam4.3933 (PMC8209574; doi:10.1002/cam4.3933)
Supplement: Supplementary file 1 — Fig S1 [file CAM4-10-3986-s004.docx]

Figure S1. Correlations between NOTCH3 and EMT markers expression in various ESCC cell lines. (A) Expression of NOTCH3 FL, ICN3, VIM, CDH1, and CDH2 in ESCC cell lines, as assessed by Western blot. ACTB served as a loading control. (B-E) Expression of *NOTCH3*, *VIM*, *CDH1*, and *CDH2* mRNA in ESCC cell lines, as assessed by reverse transcription (RT)-qPCR. *GAPDH* served as an internal control gene. NOTCH3 FL: NOTCH3 full-length, ICN3: intracellular NOTCH3.
