# Supplementary material for: NOTCH3 limits the epithelial–mesenchymal transition and predicts a favorable clinical outcome in esophageal cancer
Source: Cancer Med. 2021 May 27;10(12):3986–96. doi: 10.1002/cam4.3933 (PMC8209574; doi:10.1002/cam4.3933)
Supplement: Supplementary file 2 — Fig S2 [file CAM4-10-3986-s002.docx]

Figure S2. (A, B) Changes in cell morphology upon exposure to 20 μM 5-FU in (A) TE6 and (B) TE11 cells. Scale bar, 100 μm. (C) Flow cytometry histogram plots for CDH2 in TE11 and TE11-FR. (D, E) Cisplatin and Docetaxel sensitivity assay in TE11-FR cells.

TE11-FR: TE11 5-FU resistant cell line.
