# Supplementary material for: NOTCH3 limits the epithelial–mesenchymal transition and predicts a favorable clinical outcome in esophageal cancer
Source: Cancer Med. 2021 May 27;10(12):3986–96. doi: 10.1002/cam4.3933 (PMC8209574; doi:10.1002/cam4.3933)
Supplement: Supplementary file 3 — Fig S3 [file CAM4-10-3986-s001.docx]

Figure S3. (A) Changes in morphology of cells treated with siNOTCH3. (B) Proliferation assay of cells treated with siNOTCH3. (C) Proportion of apoptotic cells after exposure to 10 μM 5-FU for 72 hours in cells treated with siNOTCH3. (D) Changes in morphology of cells following ectopic ICN3 expression. (E) Proliferation assay of cells following ectopic ICN3 expression. (F) Proportion of apoptotic cells after exposure to 10 μM 5-FU for 72 hours following ectopic ICN3 expression. (G) Changes in morphology of cells after exposure to 20 μM 5-FU in TE11^*^ and TE11-ICN3 cells cultured in the presence of DOX.

siNOTCH3: NOTCH3 was silenced by RNA interference, TE11^*^: TE11 Tet-on control cell line, TE11-ICN3: TE11 Tet-on ICN3 cell line, DOX: Doxycycline
