# Supplementary material for: NOTCH3 limits the epithelial–mesenchymal transition and predicts a favorable clinical outcome in esophageal cancer
Source: Cancer Med. 2021 May 27;10(12):3986–96. doi: 10.1002/cam4.3933 (PMC8209574; doi:10.1002/cam4.3933)
Supplement: Supplementary file 4 — Supplementary Material [file CAM4-10-3986-s003.docx]

**ESCC cell lines**

Human ESCC cell lines TE6 (RCB1950) and TE11 (RCB2100) were obtained from the Riken Bioresource Cell Bank (Tsukuba, JPN). Cells were grown in RPMI-1640 medium (Nakalai Tesque, Kyoto, JPN) supplemented with 10% FBS (Sigma-Aldrich, St. Louis, MO, USA) in a humidified atmosphere of 5% CO_2_ at 37ºC. Cell number and viability were assessed using the Cell Counting kit-8 (Dojindo Molecular Technologies, Kumamoto, JPN) following the manufacturer’s instructions. Mycoplasma testing was routinely conducted. Cell line authentication was performed by short tandem repeat DNA profiling. TE11 derivatives expressing doxycycline-inducible ICN3, the activated form of NOTCH3 (TE11-ICN3), or an empty control vector (TE11*) have been described previously (20). ICN3 was induced by incubating cells with 1 µg/ml doxycycline (DOX, 631311, Clontech Laboratories, Mountain View, CA, USA) for 24 hours. A 5FU-resistant TE11 derivative (TE11-FR) was established by passaging TE11 cells for at least 10 times in the continuous presence of 3 µmol/L fluorouracil (5-FU, 068-01403, Wako, JPN) for more than 2 months.

**ESCC xenograft tumors**

Animal studies were performed following a protocol approved by the Ethics of Animal Experiments Committee of Osaka University. For xenograft models, TE11* and TE11-ICN3 (3.0 × 10^6^) cells were suspended in 100 μL RPMI 1640/Matrigel (Becton, Dickinson and Company, Franklin Lakes, NJ, USA) and subcutaneously injected into 8‐week‐old female mice (BALB/c‐nu/nu; CLEA Japan, Tokyo, JPN). Tumor volume was measured with calipers and calculated using the formula V = (ab^2^)/2, where a is the smallest diameter and b is the largest diameter. To induce ectopic ICN3 expression in vivo, DOX was administered to mice via drinking water (1 mg/ml in 5% sucrose) starting from Day 24 after xenograft transplantation. When the average tumor size reached 100 mm^3^, 5-FU (5 mg/kg) or PBS (vehicle control) was administered every 3 days by intraperitoneal injection for 21 days. Mice were euthanized on Day 21 and tumors were collected. The tumors were fixed in 10% buffered formalin for immunohistochemistry, or lysed for Western blot analysis.

**ESCC patients**

A total of 120 ESCC patients underwent esophagectomy following NAC at Osaka University Medical Hospital from January 2010 to December 2014 in a study performed under the Institutional Review Board-approved protocol (08226-13) in accordance with the Declaration of Helsinki. Patients received one of two regimens as NAC. The first regimen included adriamycin, cisplatin, and fluorouracil (ACF; adriamycin 35 mg/m^2^ and cisplatin 70 mg/m^2^ i.v. on Day 1, and fluorouracil 700 mg/m^2^ continuous infusion for 5 days) every 4 weeks. The second regimen included docetaxel, cisplatin, and fluorouracil (DCF; docetaxel 70 mg/m^2^ and cisplatin 70 mg/m^2^ i.v. on Day 1, and fluorouracil 700 mg/m^2^ continuous infusion for 5 days) every 3 weeks (1). Data on patient characteristics, histologic examination, and survival were obtained from medical charts. Therapeutic effect was evaluated according to the histological criteria set forth by the Japanese Society of Esophageal Disease (21). Briefly, therapeutic efficacy was divided into five categories (grade 0, 1a, 1b, 2, or 3) based on the proportion of the tumor affected by degeneration or necrosis. Patients underwent regular follow-up for 4-8 weeks after surgery, and were then assessed every 3 months during the first 2 years, every 6 months for the subsequent 3 years, and then annually from 5 years after surgery. Radiological investigations, usually by CT, were performed when there was a suspicion of recurrent disease or an endoscopic finding. All recurrences were confirmed by histological or radiological examination. Patient status was recorded at the last visit for survival analysis.

**RNA interference (RNAi)**

RNAi experiments were performed using NOTHC3-targeted siRNA and a non-silencing control sequence (siRNA‐A) purchased from Santa Cruz Biotechnology (Dallas, TX, USA). In brief, 2.5 × 10^5^ cells were seeded per well containing 2 mL antibiotic‐free RPMI 1640 medium with 10% FBS in 6‐well plates (Corning, Corning, NY, USA) and grown for 24 hours. Cells were then transfected with siRNA using the Lipofectamine RNAiMAX Transfection Reagent (Invitrogen, Carlsbad, CA, USA) according to the manufacturer’s instructions. Cell culture medium was replaced 7 hours after transfection and cells were grown for 24-48 hours prior to the 5-FU-sensitivity, migration, proliferation, and apoptosis assays described below.

**WST-8 assay**

Cell proliferation and 5-FU-sensitivity were assessed using Cell Counting kit-8 (Dojindo Molecular Technologies). In brief, cells (3.0 × 10^3^ per well) were seeded in 96-well plates (Corning), and cell proliferation was analyzed 24, 48, 72, and 96 hours later. To determine 5-FU-sensitivity, cells were grown for 24 hours in the absence of 5-FU followed by exposure to various concentrations of 5-FU (0, 0.2, 0.4, 0.8, 1.6, 3.2, 6.4, 12.8, 25.6, and 51.2 μmol/L) for an additional 72 hours to determine cell viability compared to that of 5-FU untreated cells. To determine the relative number of viable cells, cells were incubated with 10 μL Cell Counting kit-8 reagent added to each well for the last 2 hours at 37ºC, following the manufacturer’s instructions. Absorbance was measured with an iMark microplate reader (Bio‐Rad Laboratories, Hercules, CA, USA) at an excitation wavelength of 490 nm and emission wavelength of 515 nm.

**Annexin V assay**

5-FU-induced apoptotic cell death in culture was assessed by flow cytometry for Annexin V-stained cells using the Annexin V Binding Buffer (422201, Biolegend, San Diego, CA, USA). In brief, cells were stained with APC Annexin V (640920, dilution 1:20, Biolegend) and Propidium Iodide (PI) (#1056, dilution 1:40, Biovision, Milpitas, CA, USA) on ice for 10 min and at 37ºC for 15 min in the dark. Samples were analyzed by flow cytometry on a FACSVerse instrument (BD Biosciences, San Jose, CA, USA). Cells stained with Annexin V were considered apoptotic.

**Cell migration assay**

Cell migration was assessed by scratch-wound healing in monolayer culture. In brief, cells (8.0 × 10^5^ per well) were seeded in a 6-well plate (Corning) and allowed to grow for 24 hours. The confluent monolayer was then scratched using a pipette tip to create a cell-free area at time 0. Cell migration into this area was monitored for 96 hours by a microscope equipped with a camera (BZ-X710, KEYENCE, Osaka, JPN). Images were captured at 0, 24, 48, 72, and 96 hours and wound closure was analyzed by ImageJ software (37).

**Real-time quantitative PCR (qPCR)**

Total RNA was purified from sub-confluent cells using the TRI REAGENT (Molecular Research Center, Cincinnati, OH, USA) following the manufacturer’s instructions. Total RNA was quantified using Nano Drop ND-1000 (Thermo Fisher SCIENTIFIC, Waltham, MA, USA), and 1.0 μg total RNA was used to synthesize complementary DNA by reverse transcription (RT) (Reverse Transcription System; Promega, Madison, WI, USA). RT-qPCR was performed in triplicate with THUNDERBIRD^®^ SYBR^®^ qPCR Mix (TOYOBO, Osaka, JPN) and the Applied Biosystems 7900HT Fast Real Time PCR system (Thermo Fisher SCIENTIFIC). The specific primers used were as follows: *NOTCH3* (5’-GGGCTCCTTTTCCTGCTC-3’ and 5’-GGTTGCTCAGGCACTCATC-3’), *VIM* (5’-AGCTAACCAACGACAAAGCC-3’ and 5’-TCCACTTTGCGTTCAAGGTC-3’), *CDH1* (5’-GAATGACAACAAGCCCGAAT-3’ and 5’-ACCTCCATCACAGAGGTTCC-3’), and *CDH2* (5’-GGATACACAAGAGGGAGTCATCA-3’ and 5’-GGTCAAGGTGAAGGTTGGAA-3’). The relative level of mRNA for each gene was determined by the ΔΔCt method and normalized to *GAPDH* as an internal control.

**Western blot analysis**

Total protein was extracted from cultured cells using RIPA buffer containing protease inhibitors and phosphatase inhibitors (Thermo Fisher Scientific). Proteins were subjected to 12% SDS‐PAGE (Bio‐Rad, Hercules, CA, USA). The separated proteins were transferred to Immun‐Blot PVDF membranes (Bio‐Rad) and incubated with anti‐ACTB antibody (A2066, dilution 1:1000, Sigma‐Aldrich), anti‐NOTCH3 antibody (#3446, dilution 1:500, Cell Signaling Technology), anti‐CDH1 antibody (#88345, dilution 1:1000, Cell Signaling Technology), anti‐CDH2 antibody (#ab18203, dilution 1:1000, abcam, Cambridge, MA, USA), or anti‐VIM antibody (#ab8069, dilution 1:1000, abcam) at 4ºC overnight. Membranes were then incubated with HRP‐linked anti‐rabbit, mouse, or rat IgG (#7074, #7076, and #7077, Cell Signaling Technology) at a dilution of 1:10,000 or 1:100,000 for 1 hour at room temperature. The antigen-antibody complex was detected using the ECL Prime Western Blotting Detection Kit (GE Healthcare Biosciences, Menlo Park, CA, USA). In the experiments of short-term exposure to 5-FU, western blots were quantified by densitometry with Image Lab software (Bio‐Rad Laboratories). The signal intensity for molecule of interest was calibrated by that of β-actin (ACTB) at each time point (day 1-6). The relative expression was expressed compared to the signal intensity at day 1 as 1.

**Chromatin immunoprecipitation (ChIP) assay**

To perform ChIP assays, 2 × 10^6^ cells grown for 22 hours in 100-mm dishes were treated with 1% formaldehyde for 10 min at 37ºC and quenched with 0.125 M glycine for 5 min at room temperature. Cross-linked chromatin was sheared into 500 bp DNA fragments with Covaris S220 (M & S Instruments Inc. Tokyo, JPN). Sheared chromatin (20 μg) was incubated for immunoprecipitation with an antibody against NOTCH3 (#2889, dilution 1:50, Cell Signaling Technology) or RBPJ (ab25949, dilution 1:100, abcam), or negative control mouse IgG (53010, dilution 1:10, ACTIVE MOTIF, Carlsbad, CA, USA). DNA was purified with the Chromatin IP DNA purification kit (58002, ACTIVE MOTIF) and analyzed by real-time qPCR using THUNDERBIRD^®^ SYBR^®^ qPCR Mix and the Applied Biosystems 7900HT Fast Real Time PCR system (Thermo Fisher SCIENTIFIC). The following primers were used for real-time qPCR: 5’-AGCTGCAGGCGCTAGTTG-3’ and 5’-CACACCCAAACACCACGTATT-3’ for RBPJ-binding sites in the 2nd intron of *VIM*, and 5’-TTTGCCGTGATATATAGGATAATTT-3’ and 5’-TGATGCTGAGAAGTTTCGTTG-3’ for an off-target control region of *VIM*. Data represent at least three independent experiments.

**Immunohistochemistry and TdT-mediated dUTP nick end labeling (TUNEL) assay**

Tumor specimens were fixed with 10% formalin, and paraffin-embedded tissue blocks were sectioned into 3.5-μm slices. The sections were deparaffinized in xylene and dehydrated in a graded ethanol series. For antigen retrieval, sections were incubated in 10 mM citrate buffer at 110ºC using a pressure cooker for 15 min. Endogenous peroxidase activity in the tissue specimens was blocked by incubating the slides in 3% hydrogen peroxide (H_2_O_2_) solution in methanol at room temperature for 20 min. After treatment of the sections with 1% horse serum albumin for 30 min at room temperature to block nonspecific reactions, all sections were incubated with primary antibodies in a humidified chamber at 4ºC overnight. Antibodies used included anti-NOTCH3 polyclonal antibody (ab23426, dilution 1:300, abcam), anti‐CDH1 monoclonal antibody (#3195, dilution 1:100, Cell Signaling Technology), anti-CDH2 polyclonal antibody (ab18203, dilution 1:300, abcam), and anti-VIM monoclonal antibody (#5741, dilution 1:300, Cell Signaling Technology). After incubation with secondary antibodies for 20 min at room temperature, the reactions were visualized using VECTASTAIN^®^ Elite^®^ ABC Kit (PK-6100, VECTOR LABORATORIES, Burlingame, CA, USA), which stains the targeted antigen brown, and hematoxylin counterstaining. Two investigators (N. M and K. T) independently evaluated the stained sections. The grade and area of nuclear staining with the anti-NOTCH3 antibody of cells remaining after NAC were evaluated and divided into two groups: ICN3-positive and ICN3-negative. The degree of CDH2 and VIM staining was evaluated based on the extent of membranous staining.

TUNEL assays were performed to evaluate apoptosis in formalin-fixed, paraffin-embedded xenograft tumor tissue samples. In brief, paraffin sections (3.5 μm) were deparaffinized with xylene and then rehydrated in a graded alcohol series. TUNEL signal was detected using the ApopTag Fluorescein In Situ Apoptosis Detection Kit (Chemicon International, Temecula, CA, USA). Nuclei were counterstained using VECSTASHIELD Mounting Medium with DAPI (VECTOR Laboratories). Green fluorescence from apoptotic cells was analyzed with a fluorescence microscope (BZ-X 710; KEYENCE, Osaka, JPN). TUNEL-positive cells were considered apoptotic cells.

**Statistical Analysis**

Each experiment was repeated three times. Data are expressed as mean ± SD. Mean values were compared using Student’s t-test. In vivo tumor growth was analyzed with one-way ANOVA for repeated measures. Discrete variables were assessed with the χ^2^‐test. Overall survival (OS) was defined as the time interval between the day of surgery and day of death or last follow-up. Recurrence-free survival (RFS) was defined as the time interval between the day of surgery and documented date of first recurrence. Survival was calculated according to the Kaplan–Meier method and compared by the log rank test. P<0.05 was considered statistically significant. Statistical analyses were performed using JMP Pro 14.0 (SAS Institute, Cary, NC, USA).
